# Suppressing NRIP1 inhibits growth of breast cancer cells *in vitro* and *in vivo*

**DOI:** 10.18632/oncotarget.5356

**Published:** 2015-10-15

**Authors:** Moammir H. Aziz, Xundi Chen, Qi Zhang, Chad DeFrain, Jared Osland, Yizhou Luo, Xin Shi, Rong Yuan

**Affiliations:** ^1^ Division of Geriatrics, Department of Internal Medicine, Southern Illinois University School of Medicine, Springfield, Illinois 62794-9628, USA; ^2^ Department of Medical Microbiology and Immunology and Cell Biology, Southern Illinois University School of Medicine, Springfield, Illinois 62794-9628, USA; ^3^ Zhongda Hospital, Southeast University of China, Nanjing, China 210009, USA; ^4^ Department of Pathology, Southern Illinois University School of Medicine, Springfield, Illinois 62794-9628, USA; ^5^ Department of Oncology, Nanjing Junxie Hospital, Nanjing, China, 210002

**Keywords:** NRIP1, breast cancer, human tissue array, apoptosis, cell survival

## Abstract

Earlier age at menarche is a major risk factor for breast cancer. Our previous study identified *Nrip1* (also known as *Rip140*) as a candidate gene for delaying female sexual maturation (FSM) and found that knocking out *Nrip1* could significantly delay FSM in mice. To investigate the effects of NRIP1 in breast cancer we used human cell lines and tissue arrays along with an *in vivo* study of DMBA-induced carcinogenesis in *Nrip1* knockout mice. Analysis of tissue arrays found that NRIP1 is elevated in tumors compared to cancer adjacent normal tissue. Interestingly, in benign tumors NRIP1 levels are higher in the cytosol of stromal cells, but NRIP1 levels are higher in the nuclei of epithelial cells in malignancies. We also found overexpression of *NRIP1* in breast cancer cell lines, and that suppression of *NRIP1* by siRNA in these cells significantly induced apoptosis and inhibited cell growth. Furthermore, *in vivo* data suggests that NRIP1 is upregulated in DMBA-induced breast cancer. Importantly, we found that DMBA-induced carcinogenesis is suppressed in *Nrip1* knockdown mice. These findings suggest that NRIP1 plays a critical role in promoting the progression and development of breast cancer and that it may be a potential therapeutic target for the new breast cancer treatments.

## INTRODUCTION

An epidemiologic study of the human population found that earlier age at menarche (AAM) significantly associates with the risk of breast cancer [1]. Remarkably, a 5-year delay in menarche has been shown to correspond to a 35% reduction of breast cancer risk [2], suggesting that genes that accelerate female sexual maturation may be involved in promoting tumorigenesis in breast tissue. However, the underlying genetic and molecular mechanisms remain unclear. By investigating the genes that regulate female sexual maturation (FSM), we hope to provide clues for the identification of novel breast cancer genes and possible therapeutic targets.

Previous studies found that NRIP1 (also known as RIP140) could regulate age of female sexual maturation and the development of mammary gland in mice [3, 4]. Importantly, a human genome-wide association study found that *NRIP1* is significantly associated with the risk of breast cancer [5]. Despite this increasing evidence for the role of NRIP1 in the progression and development of cancer [4 – 15], the mechanisms are poorly understood. Specifically in relation to breast cancer, NRIP1 was found to have higher level in luminal-like breast cancer than in basal-like tumors [6]. In addition, both *in vitro* and *in vivo* studies suggest that the E2F pathway exerts direct transcriptional control on NRIP1 expression [6, 7]. This regulation may play an important role in gene transcription and cell proliferation, differentiation, growth and apoptosis, which are strongly associated with the breast tumor development and progression.

In order to evaluate if NRIP1 influences cell growth, apoptosis and progression of breast cancer, we used human breast cancer cell lines and human breast cancer tissue arrays along with *in vivo* experiments using *Nrip1* deficient mice. Our results indicate that *NRIP1* was overexpressed in human breast cancer tissue and cell lines. The suppression of NRIP1 in human cancer cells using siRNA may induce apoptosis and inhibit cell growth. Our results further suggest that 7,12-dimethylbenz[a]anthracene (DMBA) treatment caused up-regulation of *Nrip1* in breast cancer tissue from wildtype mice. Importantly, we found that DMBA-induced carcinogenesis is suppressed in *Nrip1* deficient mice. Taken together, our current experimental data suggest that NRIP1 plays an important role in the development of breast cancer and it may be a novel therapeutic target for the treatment of breast cancer.

## RESULTS

### Suppressing the over-expression of *NRIP1* in human breast cancer cells inhibits cell growth and induces apoptosis

#### NRIP1 expression

**Figure 1:**
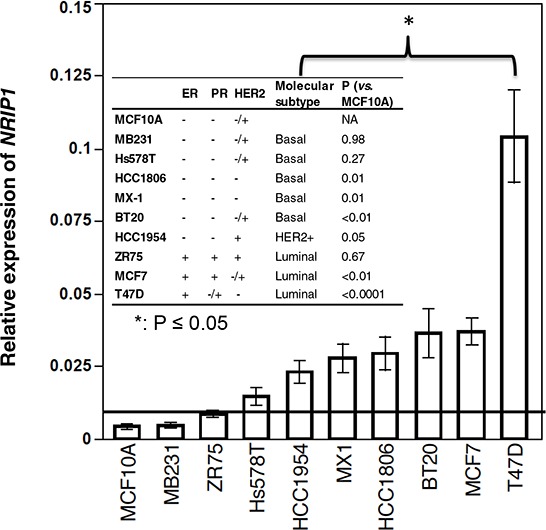
NRIP1 expression elevated in most breast cancer cell lines Expression of *NRIP1* was measured by real-time PCR in RNA isolated from breast cancer cell lines. Expression of GAPDH mRNA was used as an internal control. MCF10A was used as a normal control. The horizontal bar indicates the 2-fold change compared to MCF10A. Bars on each column indicate the standard error (*n* ≥ 3). The *p* values of the t test between cancer cell lines *vs.* MCF10A are listed in the table. *: *t*-test, *p* ≤ 0.05.

At the time of clinical diagnosis, breast cancers can present as a wide variety of subtypes based on histopathological, biological and molecular characteristics [16–19]. Therefore, recognition of the specific oncogene to target all cancer subtypes seems to be an effective approach for breast cancer management. In order to better understand the importance of NRIP1 in human breast cancers, we first evaluated the expression of *NRIP1* mRNA using real-time PCR in three luminal cell lines (ZR75, MCF7, and T47D), five basal or triple negative lines (HCC1806, MX-1, BT20, Hs578T and MDA-MB-231) and one HER2+ line (HCC1954). One immortalized line (MCF10A) was used as a control. Compared to MCF10A, *NRIP1* mRNA levels were elevated in all nine cell lines. The elevation is more than a 2 fold-change (FC) in every line except MDA-MB-231. All cell lines except MDA-MB-231, ZR75 and Hs578T had significantly higher *NRIP1* expression (*t*-test, *P* ≤ 0.05). The highest level of *NRIP1* mRNA expression was found in T47D cells, and was 28 times higher than that in MCF10A cells (Fig. 1). These results were further confirmed by immunofluorescence (S. Fig. S1).

#### NRIP1 depletion

By using siRNA targeting *NRIP1* (siNRIP1), we suppressed *NRIP1* expression in 5 breast cancer cell lines (MCF7, T47D, HCC1806, MDA-MB-231 and HCC1954), which includes all three molecular subtypes of breast cancer, and used MCF10A as a control. The effectiveness of *NRIP1* inhibition was first examined by real-time PCR (S. Fig. S2A) at 24, 48 and 72 h after the treatment. We detected maximum *NRIP1* mRNA inhibition (~87%) in T47D at 72 h and minimum inhibition (~55%) in HCC1806 at 48 h compared to their respective nonsense siRNA (siCON) treated controls. Inhibition of NRIP1 expression was further confirmed by western blot analysis (S. Fig. S2B).

**Figure 2:**
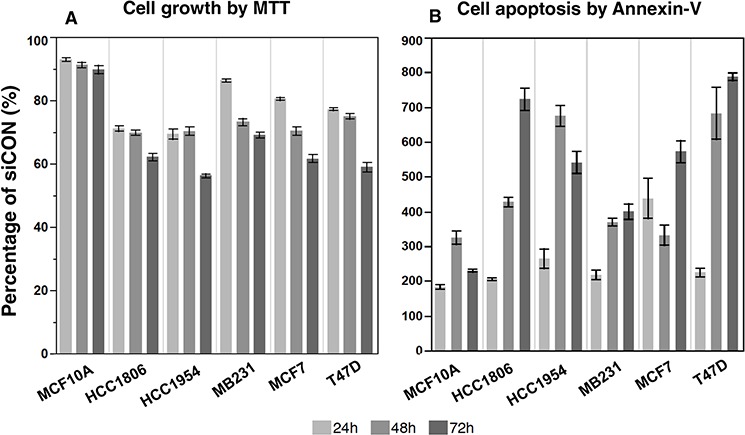
The suppression of NRIP1 expression inhibits cancer cell growth and induces apoptosis After the siRNA treatment, the cell growth and apoptosis were measured by MTT **A.** and Annexin-V **B.** assays respectively. At all time points and in all cells, the siNRIP1 significantly suppresses cell growth and induces apoptosis compared to the siCON treated cells (*p* < 0.05), except the apoptosis in MCF10A at 24 and 72 h (*p* = 0.07 and 0.06 respectively).

These differences in siRNA suppression efficiencies of NRIP1 expression may be due to the fact that NRIP1 expression varies dramatically among the cell lines (Fig. 1, S. Fig. S1 and 2B) and these cell lines have different growth rates (Fig. 2A). Furthermore, the effect of transfection on *NRIP1* in the current study is not stable, contributing to variation of knockdown efficiencies among cell lines and time-points.

#### Cell growth

Employing the MTT assay, siNRIP1 reduced cell growth in all cell lines compared to siCON cells (Fig. 2A). The reductions were significant in all cell lines and at all time points (*P* < 0.05). The most significant reduction was found in HCC1954 at 72 h after the treatment, in which the growth of siNRIP1 treated cells was reduced to 44% of the siCON treated cells. However, the siNRIP1 treated MCF10A had the lowest reduction at all time-points.

#### Cell cycle

To investigate the underlying reasons for the reduced cell growth after suppressing *NRIP1*, we further examined cell proliferation and apoptosis in siNRIP1 treated cells by propidium iodide (PI) staining assay and flow cytometry at 24, 48 and 72 h after treatment (S. Fig. S1A–1C). The percentage of G0/G1 cells significantly decreased (*P* < 0.05) at all three time points in breast cancer cell lines except MCF10A cells and T47D cells. At 24 and 48 h, the reductions were significant across the cell lines (overall *P* < 0.05). Interestingly, the percentages of G0/G1 in T47D and MCF10A cells increased at 72 h (*P* = 0.07 and 0.02 respectively). No clear trend of alteration by siNRIP1 treatment in S and G2/M phases could be found across the cell lines.

#### Apoptosis induction

The induction of apoptosis by the NRIP1 suppression was striking. PI staining indicated a significant induction of apoptosis in all cells lines at every time point (*P* < 0.05, S. Fig. S1A–1C), except MCF10A at 72 h after siNRIP1 treatment. To verify this result, we used the Annexin-V assay to detect apoptotic cells. Similar to PI staining, the induction of apoptosis was significant in breast cancer cells at each time point (Fig. 2B, Supplementary Table 2), except MCF10A at 24 and 72 h (*P* = 0.07 and 0.06 respectively). It is worth mentioning that both PI staining and Annexin-V assay found that siNRIP1 treatment could significantly induce apoptosis at all three time points across the cell lines (*P* < 0.05, S. Fig. S1–S2). These results suggest that NRIP1 is required for cell growth and anti-apoptotic activities in breast cells.

### Elevated NRIP1 expression in human breast cancer

To determine the clinical significance of NRIP1 in breast cancer, we analyzed tissue arrays (US Biomax, Rockville, MD) containing clinical specimens from 75 cases/150 cores, including 138 malignant cores, 6 benign cores and 6 cancer adjacent normal tissue (CANT) cores. Information regarding molecular markers for each case, such as Her2, ER, PR, p53, and Ki67, was provided by the manufacturer. According to molecular markers, triple negative (basal), luminal and HER2+ subtypes were represented by 60, 56 and 22 cores, respectively.

The NRIP1 stained sections were digitally imaged using a slide scanner (US Biomax, Rockville, MD) for further analysis. The staining of NRIP1 was then assessed in 10 microscopic fields (40x) for each core, in a blinded fashion by two individuals, including one clinical pathologist (Dr. DeFrain). To assess the general level of NRIP1, the staining was scored between 0 and 3, with scores indicating positive NRIP1 staining of 0–5%, 6–25%, 26–50% and 51–100% respectively. We also assessed the subcellular level of NRIP1 in specific cells in the tissues. In each field, the total number of epithelial and stromal cells, as well as the numbers of cells with positive staining nuclear and cytoplasm were counted. Then the percentages of positive staining at subcellular levels in different types of cells were calculated. The intensity of the staining in the subcellular location was scored from 1 to 3. The NRIP1 subcellular level is calculated by using formula: intensity × percentage of positive staining. The scores were then correlated with clinical parameters.

**Figure 3:**
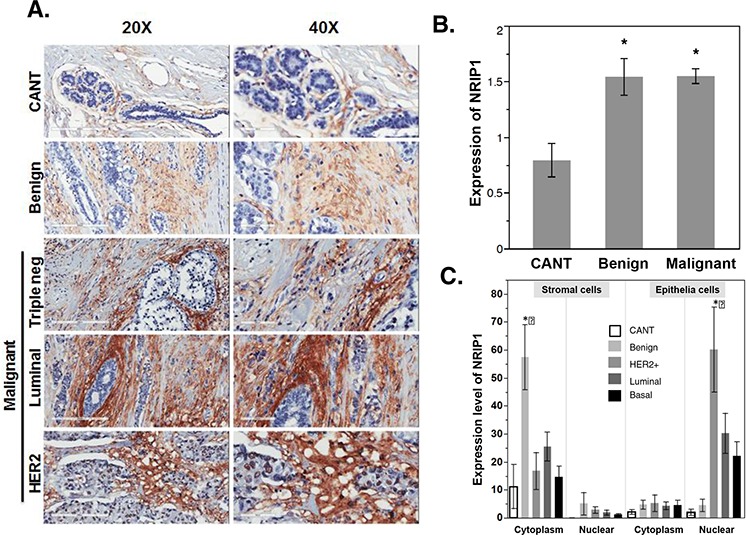
NRIP1 overexpression in human cancer tissue **A.** Immunohistochemical staining for NRIP1 expression was shown in cancer adjacent normal tissue (CANT), benign breast and malignant breast tissue (HER2, Luminal & Triple negative). Representative images, magnification [20X (left panel) and 40X (right panel)] and scale bar [200 μm (left panel) and 100 μm (right panel). **B.** Quantitation of NRIP1+ cells in the CANT, Benign and malignant human breast cancer tissue. **C.** Quantitation of NRIP1+ cells in stromal (cytoplasm and nuclear) and epithelia (cytoplasm and nuclear) regions of CANT, Benign, HER2+, Luminal and basal area of breast tissue.

Analysis of the general immunostaining score indicated that benign and malignant tumors had significantly higher NRIP1 expression levels than CANT (*P* < 0.001, Fig. 3). Further analysis found that among the three malignant tumor subtypes, there was no significant difference in NRIP1 levels; however, NRIP1 levels were elevated in each of the subtypes compared to CANT (Table 1). Correlation analysis found that NRIP1 positively correlated with PR (*P* = 0.03). No significant correlation between the clinical grades and levels of NRIP1 was found.

**Table 1:** Score of NRIP1 expression in different type of breast cancers and adjacent normal tissue

	Mean	STE	No.	P*
**Luminal**	1.7	0.1	56	0.01
**HER2**	1.6	0.2	22	0.03
**Basal**	1.4	0.2	60	0.06
**CANT**	0.8	0.2	6	-

* compared to the CANT

Expression of NRIP1 was primarily limited to the periductal stroma in CANT and benign tumors, while malignant tumors had widespread staining in stromal and epithelial cells. Periductal stroma of benign tumors showed significantly increased NRIP1 expression in the cytoplasm compared to CANT (*P* < 0.05, Fig. 3A, 3C). Occasional nuclear staining was seen in the terminal duct lobular unit of benign and malignant tumors, but no nuclear staining was seen in the stromal cells in CANT. In epithelial cells of CANT and benign tumors, faint staining was seen in both the cytoplasm and nucleus. However, malignant tumors had elevated nuclear staining intensity in the epithelium (Fig. 3A, 3C). Nuclear NRIP1 levels in the epithelium of HER2+ tumors were 15 and 30 times higher than benign tumors and CANT, respectively. Compared to CANT, the elevation is significant (*P* < 0.05).

### Depletion of *Nrip1* decreased susceptibility of DMBA-induced mammary

**Figure 4:**
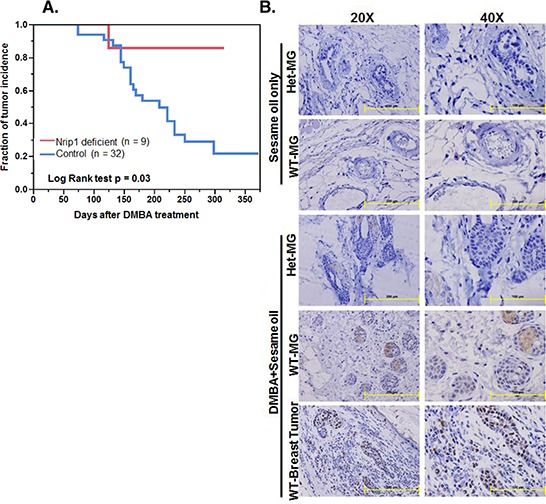
Reduced NRIP1 expression results in decreased susceptibility to DMBA-induced mammary tumor incidence in mice Sesame oil only and DMBA treated WT (control) and knockdown (*Nrip1* deficient) mice were examined weekly for palpable tumors and **A.** All tumors incidence of DMBA treated WT and *Nrip1* deficient mice were recorded and figure represents the days until first palpable tumor was detected. **B.** Immunohistochemical staining for NRIP1. Normal breast and breast tumor specimens were fixed in the formalin for immunohistochemical study. Expression of NRIP1 in sesame oil only treated mice [Wildtype-Mammary Galnd (WT-MG) and Heterozygous-Mammary Gland (Het-MG)] and DMBA+sesame oil treated mice [Wildtype-Breast tumor (WT-Breast Tumor), WT-MG and Het-MG] specimens. Representative images, magnification [20X (left panel) and 40X (right panel)] and scale bar [200 μm (left panel) and 100 μm (right panel).

The elevated NRIP1 levels in cancer cell lines and tumor samples, as well as the induction of apoptosis by siNRIP1 *in vitro*, suggested that the depletion of NRIP1 might suppress the breast tumors *in vivo*. To test this hypothesis, we investigated the specific role of NRIP1 in breast cancer growth and development using a 7,12-dimethylbenz[a]anthracene (DMBA)–induced mouse cancer model using female wildtype (*n* = 32) and *Nrip1* deficient mice (*n* = 9), including both heterozygous (*n* = 7) and homozygous knockouts (*n* = 2). Mice were given 6 weekly 1 mg doses of DMBA in 200 μl of sesame oil by oral gavage, beginning at 6 weeks of age. Vehicle control mice were given sesame oil (200 μl) only. DMBA treated wildtype mice began to develop tumors at 11 weeks. Of the 32 wildtype mice, 21 had developed tumors 40 weeks after DMBA treatment (Fig. 4). Among those 21 mice, we found 5 mice with breast tumors, 14 mice with skin tumors, 3 mice with liver tumors, and 3 mice with intestinal tumors. However, with the exception of one skin tumor, no malignancies were found among the nine *Nrip1* deficient females. Additionally, the skin tumor in the *Nrip1* deficient mouse was smaller than the skin tumors found in the wildtype mice. The log-rank test of the tumor incidence fraction curves found the difference between the wildtype and *Nrip1* deficient mice was significant (Fig. 4A, *P* = 0.03). The tumor risk was also significantly lower in the *Nrip1* deficient mice based on the chi-square test, *P* < 0.001.

The *Nrip1* expression level was determined by immunohistochemical analysis. Mammary glands from control mice of both groups (wildtype and *Nrip1* deficient) exhibited no immunoreactivity to the anti-NRIP1 antibody in the stromal or epithelial areas. Slight immunostaining was found in the cytoplasm of epithelial cells (~5%) of normal mammary gland tissue collected from DMBA treated *Nrip1* heterozygous knockout mice. In normal mammary gland tissue of DMBA treated wildtype mice, moderate staining was found in the cytoplasm and nucleus of the epithelial cells (~15%). Elevated NRIP1 expression was observed in mammary gland tumors collected from DMBA treated wildtype mice. Intensive staining was spread throughout the cytoplasmic and nuclear regions of both epithelial and stromal cells (>50%) (Fig. 4B).

## DISCUSSION

NRIP1 is a co-regulator for various nuclear receptors and transcription factors shown to be involved in various human cancers [4, 6–13]. Previous studies suggests that *Nrip1* expression is associated with mammary gland development in mice via activation of ER signaling as well as a number of regulatory genes (e.g. *Arg, Pgr* and *Stat5a*) [4]. In the current study, we investigated the role of NRIP1 in breast cancer development in order to elucidate the effect of NRIP1 on cell growth, apoptosis and cell cycle arrest. Our study reveals that NRIP1 level is elevated in human breast cancer cell lines compared to normal immortalized epithelial cells (MCF10A). Luminal cancer cell lines, such as T47D and MCF7, have the highest expression level, and the basal-like cancer cell line MDA-MB-231 had the lowest expression level. However, the other basal cell lines (HCC-1806, MX-1, BT20 and Hs578T) had relatively higher *NRIP1* expression than that of luminal cell line ZR75. It has been previously reported that estrogen signaling could up-regulate *NRIP1* expression in MCF-7 cells [14]. However, the current results found elevated *NRIP1* expression in basal type tumors, which are ER negative, suggests that other mechanisms may also be involved in regulating *NRIP1* expression. Indeed, *NRIP1* is a target gene of microRNA-125b (miR-125b), which has been found to be underexpressed in ductal carcinomas [15]. Thus, *NRIP1* overexpression in breast cancers might also be related to the dysregulation of miR-125b.

Chip-seq analysis found that NRIP1 plays an essential role in the formation of the ERβ complex and in the regulation of ERα downstream gene expression [8]. Rosell M. *et al.* further demonstrated that suppressing NRIP1 in ER positive MCF7 cells could inhibit estrogen promoted cell proliferation [8]. Our results with siNRIP1 treated ER positive cells further supports the role of NRIP1 in ER mediated cell growth. Interestingly, the cell growth of ER and HER2 negative HCC1806 breast cancer cell line was also clearly attenuated by siNRIP1, suggesting the involvement of other signaling pathways in siNRIP1 suppressed cell growth in addition to the activation of ER. To further confirm whether the cell growth and growth was induced through apoptosis induction, cell cycle and apoptosis analyses were performed using PI staining and annexin v assays demonstrated that targeting NRIP1 in breast cancer cells significantly induced apoptosis irrespective of breast cancer subtypes, suggesting that the suppression of cell growth and growth is due to the induction of apoptotic cell death. This result provides evidence at the cellular level to explain a recent observation that the distant metastasis-free survival has a significant correlation with NRIP1 activity [8, 20].

The immunohistochemical assay found that NRIP1 level is elevated both in benign and malignant breast tumors compared to normal tissue adjacent to the tumors. This result is in agreement with previous human breast cancer tissue microarray findings that NRIP1 expression was significantly overexpressed in luminal tumors than in basal like tumors [6]. In our current report, we also found evidence at the protein level that luminal cancers have higher NRIP1 expression than basal cancers (Table 1), while the NRIP1 level in HER2 positive cancers is higher than basal cancer but lower than the luminal cancers. Our *in vivo* data further suggest that DMBA-induced breast cancer incidence is significantly suppressed in *Nrip1* deficient mice compared to wildtype mice. Combined, the human breast cancer tissue array results and the DMBA induced mammary tumor data (Fig. 4) strongly indicates that *NRIP1* upregulation is related to breast cancer progression. Interestingly, *Nrip1* was overexpressed in normal mammary tissue from DMBA treated wildtype mice, indicating that NRIP1 is involved in early stages of the progression of breast cancer.

Previous studies have suggested that post translational modifications (PTMs) of NRIP1 include phosphorylation, acetylation, methylation, PLP conjugation, ubiquitination, and sumoylation [21–23]. These modifications can modulate gene regulatory activity, protein stability, cellular distribution, and/or interacting partners [19]. These PTMs can be categorized into two principle pathways, stimulating cytoplasmic activities of NRIP1 through increased nuclear export and enhancing the gene regulatory activities of NRIP1 through increased nuclear retention [23]. Our present study highlights the subcellular distribution of NRIP1 expression in stromal cells and epithelial cells. The pattern of NRIP1 expression in breast cancer suggests that NRIP1 expression is associated with stromal cells as well as epithelial cells. However our study further suggests that the distribution of NRIP1 is different at both the subcellular level (cytosolic and nuclear regions) and the cellular level (epithelial and stromal cells). In stromal cells, NRIP1 is overexpressed in the cytosol and in epithelial cells the expression is higher in nucleus. These results suggest that the subcellular localization of activated NRIP1 plays a significant role in breast cancer progression, however the mechanism needs to be thoroughly investigated.

Several earlier studies support our recent findings that NRIP1 plays an important role in breast cancer and its development [4, 6–9, 15]. However, a recent study demonstrated a decrease in NRIP1 expression at both transcriptional and translational levels in human colon cancers, which contradicts our recent findings [23]. This study suggests that low NRIP1 expression in adenocarcinomas is correlated with poor prognosis. Furthermore, the authors reported *in vitro* and *in vivo* data indicating that overexpression of NRIP1 inhibits intestinal epithelial cell progression and cell proliferation [24]. These contradictory effects of NRIP1 indicate the complicated role of NRIP1 on cell growth and tumorigenesis in different tissues.

## CONCLUSION

Our present study suggests that NRIP1 has an important role in cell growth and apoptosis irrespective of cancer types, is associated with luminal, HER2 and basal grade breast carcinomas and is also involved in the progression of chemically induced breast carcinogenesis. This suggests that NRIP1 may be a novel therapeutic target for the treatment of breast cancer. However, further detailed studies are needed to validate the specific mechanism of NRIP1 in various types of human breast cancer and its clinical relevance.

## MATERIALS AND METHODS

### Chemicals, antibody, and assay kits

7,12-dimethylbenz[a]anthracene (DMBA; Sigma-Aldrich; purity: ≥95%), NRIP1 antibody used in this study was purchased from Santa Cruz, Biotechnology (Santa Cruz, USA), fetal bovine serum (FBS; Atlanta Biologicals, Inc., Flowery Branch, GA); PE Annexin-V Apoptosis Detection Kit (BD Pharmingen); propidium iodide (25 μg/ml; Calbiochem); Dimethyl sulfoxide (Fisher Scientific, USA), Tween 20 (ACROS, New Jersey, USA). The forward and reverse primers for NRIP1, and GAPDH were custom synthesized from IDT technology (Integrated DNA Technology, Coralville, IA, USA) and are listed in the RT-qPCR section. SensiFAST SYBR Hi-ROX Kit (Bioline USA Inc., USA).

### Animals

*Nrip1* KO mice were obtained from Dr. M. Parker [25]. Heterozygous (het KO) females and males were used as breeders. 6–17 weeks old *Nrip1*-Het/Hom KO (*n* = 9) and wildtype (*n* = 32) virgin female mice were housed in environmentally controlled chambers that were maintained on a 12:12-h light:dark cycle at 22 + 1°C with 35% to 50% relative humidity. They were allowed access to food and sterilized water *ad libitum*; throughout the experiment, animal care and handling were conducted in accordance with NIH guidelines and the policies of Southern Illinois University School of Medicine Laboratory Animal Care and Use Committee, Springfield, IL.

### DMBA treatment

In our NRIP1 knockdown mouse model study, we used 7,12-dimethylbenz(α)anthracene, (DMBA), a polycyclic aromatic hydrocarbon, to induce tumors in mice, a classic mouse model of breast cancer development that has been used for several decades [26, 27]. The body weight, tumor volume, survival analysis, number of mice with palpable tumors, tumor size, and number of tumors per mouse were recorded every third day for the duration of the study. Mice bearing tumors >0.5 cm were euthanized and samples were collected for further study. All the surviving mice in both groups were sacrificed at the end of experiment (13 months post DMBA treatment), followed by necropsy. The remaining tissues were collected in tubes and snap-frozen by submerging the tubes in a mixture of dry ice and ethanol. Frozen tissues were stored at −80°C.

### Histology

Mammary tumors and normal mammary gland specimens were excised and fixed overnight in 10% neutral buffered formalin, transferred to PBS (pH 7.4), and then embedded in paraffin. Sections (4 μm thick) of each specimen were cut for immunohistochemical study.

### Cell lines

Human breast cancer cell lines (BT-20, HCC1806, HCC-1954, HS-578T, MCF-7, MDA-MB-231, MX-1, T47D, ZR-75–1) and normal immortalized breast cells (MCF10A) were purchased from ATCC (American Type Culture Collection, Manassas, VA, US 20108). All the cells were grown in Dulbecco modified Eagle's medium (DMEM) supplemented with 100 U/ml penicillin, 100 μg/ml streptomycin (*CellgroMediatech*, Herndon, VA), and 10% FBS, except MCF10A cells, which were grown in Mammary Epithelial Cell Medium (MEGM; *MEGM Bullet Kit*, *Lonza Corporation*, *Walkersville*, MD, USA).

### Immunofluorescence

Breast cancer cells (HCC1806, HCC-1954, MCF-7, MDA-MB-231, T47D, ZR-75–1) and normal immortalized breast cells (MCF10A) were plated on glass coverslips in 6 well dishes. After 24 h, cells were fixed in 4% paraformaldehyde. Coverslips were next blocked and permeabilized in 1% bovine serum albumin (BSA), 0.1% Triton X-100 in PBS and then incubated with NRIP1 antibody (SCBT, Santa Cruz, CA) conjugated with CF^TM^633 (Sigma Aldrich, St. Louis, MO; 1:50 dilution in PBS/1% BSA) for overnight at 4°C. Cells were mounted with coverslip with a drop of mounting medium with DAPI. Fluorescent staining was visualized using fluorescence microscope (Olympus, Center Valley, PA, USA).

### NRIP1 siRNA transfection

The transfection was done as per the manufacturer's instructions (Santa Cruz, CA). Nontargeting siRNA Control-A (siCON) was used as a control and *NRIP1*-specific siRNA (siRNA) was used to silence *NRIP1*. The selected human breast cancer cell lines and normal mammary epithelial cell line were seeded at a density of 2 × 10^5^ cells/well in six-well plates with 2 ml complete medium without antibiotics per well. After 18 to 24 h of incubation, the cells were transfected with siRNA or siCON in medium with serum and antibiotics. The siRNA or siCON were mixed with transfecting reagent (Lipofectamine 3000, Life Technologies, Grand Island, NY) and incubated for 45 min at room temperature. The siRNA transfection reagent mixture was gently mixed and then overlaid onto the cells and incubated in 1 ml of the medium without serum or antibiotics for 6 h, after which 1 ml of normal medium containing 2 times the normal serum and antibiotics concentration (2x normal growth medium) was added without removing the transfection mixture. After 18–24 h, the medium was replaced with fresh 1x normal growth medium. After 24, 48 and 72 h of siRNA transfection, the cells were harvested for qRT-PCR, MTT assay, PE Annexin-V apoptosis assay and PI staining.

### Western blot analysis

Whole cell extracts were prepared by resuspending cell pellets in M-PER Mammalian protein extraction reagent (Thermo Scientific, Rockford, IL), resolved by sodium dodecyl sulfate polyacrylamide gel electrophoresis (SDS-PAGE) and transferred to polyvinylidene difluoride membranes (NEN Life Science Products). Antibodies to NRIP1, GAPDH were purchased from Santa Cruz Biotechnology. Primary antibodies were detected with goat anti-mouse secondary antibodies conjugated to horseradish peroxidase (Life Technologies, Carlsbad, CA), using enhanced chemiluminescence (Thermo Scientific, Rockford, IL).

### Real time quantitative polymerase chain reaction (RT-qPCR)

Total RNA was isolated by TRIzol Reagent (Invitrogen, Carlsbad, CA) using the manufacturer's single-step chloroform-extraction protocol. Complementary DNA was synthesized using the Verso cDNA synthesis kit (Thermo Scientific, USA). An aliquot of 1 μg of total RNA was reverse-transcribed by reverse transcriptase using anchored Oligo dT primers according to the manufacturer's instructions. Real-time PCR was performed using the SensiFAST SYBR Hi-ROX Kit (Bioline USA Inc., USA) on the ABI StepOnePlus Real-Time PCR machine (Applied Biosystems, Foster City, CA). The reaction system of RT-qPCR was: 10 μl of 2 × SensiFAST SYBR Hi-ROX Mix, 0.8 μl of 10 μM for each primer, 1 μl cDNA and 7.4 μl nuclease-free distilled water. Reaction parameters were: 95°C for 2 min, then 95°C for 5 s and 60°C for 15s, 40 cycles. For hNRIP1, the forward primer sequence was 5′-GCTGGGCATAATGAAGAGGA-3′, and the reverse primer sequence was 5′-CAAAGAGGCCAGT AATGTGCTATC-3′. For hGAPDH, the forward primer sequence was 5′-ATGGGGAAGGTGAAGGTCG-3′, and the reverse primer sequence was 5′-GGGGTCAT TGATGGCAACAATA-3′. Relative gene expression of *NRIP1* was calculated with the 2^−(ββCT)^ method, using GAPDH as the reference gene. Real Time PCR assays were repeated 3 times.

### Cell growth assay

The cells were plated at a density of 250 × 10^3^ cells per well in 1000 μL of complete medium without antibiotics in 6-well plates for 18–24 hrs. The cells were divided into two groups: 1) control siRNA group; 2) NRIP1 siRNA group. The transfection of siRNAs was done the following day as described previously. Each group was repeated in 2wells. The rate of cellular proliferation was measured every 24, 48 and 72 h post siRNAs transfection. At the end of each time point, 100 μL of 5 mg/ml 3-(4,5)-dimethylthiazol-2-yl-2,5-diphenyltetrazolium bromide (MTT, Sigma, St. Louis, MO) was added to each well. Four hours later, aspirate the media and 1 mL of DMSO was added to the MTT-treated wells. Each group was divided into 10 wells of a 96 well microtiter plate before determining the absorption at 540 nm by spectrophotometry (BioTek PowerWave XS, Winooski, VT, US).

### Propidium iodide (PI) staining

For cell cycle and apoptosis analysis, cells were transfected with siRNA as described above, then trypsinized and resuspended in PBS with 0.1% bovine serum albumin. A total of 1 × 10^6^ cells per mL were fixed in 25% ethanol overnight at 4°C. The cells were then stained with PI (50 mg/mL) containing RNase A (0.7 mg/mL), and incubated at 37°C. For each sample, 10,000 events were collected and examined by flow cytometry (BD Accuri C6, Franklin Lakes, NJ). Apoptosis of each group was assayed three times.

### Annexin-V staining

For cell apoptosis detection, the cells were plated in 6-well plates and were divided into the same two groups as in the MTT assay. The siRNA transfection was as before. After 24, 48 and 72 h, cells were collected for apoptosis assay and examined by flow cytometry (BD Accuri C6). The cells were stained with PE Annexin-V and 7-Amino-Actinomycin (7-AAD) following the manufacturer's instructions (BD Pharmingen, Franklin Lakes, NJ) to detect early apoptosis cells (PE Annexin-V^+^/7-AAD^−^ events) and late apoptosis cells (PE Annexin-V^+^/7-AAD^+^ events). Apoptosis of each group was assayed three times.

### Immunohistochemistry (IHC) with tissue array slides

Breast cancer tissue array slide with cancer adjacent normal breast tissue (75 cases/150 cores), was purchased (US Biomax, Inc., Rockville, MD) and used to detect the expression of the NRIP1 protein. The array included information on TNM (tumor, lymph node, metastasis) classification, clinical stage and pathology grade, with IHC results for Her-2, ER, PR, p53, AR and Ki67, and contained 3 cases/6 cores of cancer adjacent normal breast tissue as controls. Tissue array slides were deparaffinized with xylene and rehydrated with decreasing ethanol concentrations. Antigen retrieval was performed by boiling-bath method in 0.01 M sodium citrate buffer, pH 6.0 to about 95°C, and then put array slides in the buffer for 15 min. Blocking solution was used to prevent nonspecific binding of antibodies. The sections were incubated with polyclonal anti-NRIP1 antibody (SCBT, Santa Cruz, CA, 1:50 dilution) overnight at 4°C. HRP Detection System (Dako EnVision System HRP; Dako North America, Inc. Carpinteria, CA), was used for detection. After counterstaining with hematoxylin (Harris Modified Hematoxylin, Fisher Scientific, Fairlawn, New Jersey), the sections were dehydrated and mounted. The specific staining of NRIP1 in the sections was examined microscopically (Olympus, Center Valley, PA, USA).

### Statistical analysis

The data were presented as mean ± standard errors (S.E.) and examined for their statistical significance of difference was performed by using Student's *t*-test. *P*-values of less than 0.05 were considered statistically significant. Log-Rank test was used to determine the statistic significance of the fraction of tumor incidence. All statistical analyses were conducted by using JMP 10 (SAS Ins. Cary, NC).

## SUPPLEMENTARY FIGURES AND TABLES


