# Supplementary material for: Suppressing NRIP1 inhibits growth of breast cancer cells in vitro and in vivo
Source: Oncotarget. 2015 Oct 15;6(37):39714–24. doi: 10.18632/oncotarget.5356 (PMC4741857; doi:10.18632/oncotarget.5356)
Supplement: Supplementary file 1 [file oncotarget-06-39714-s001.pdf]

SUPPLEMENTARY FIGURES AND TABLES

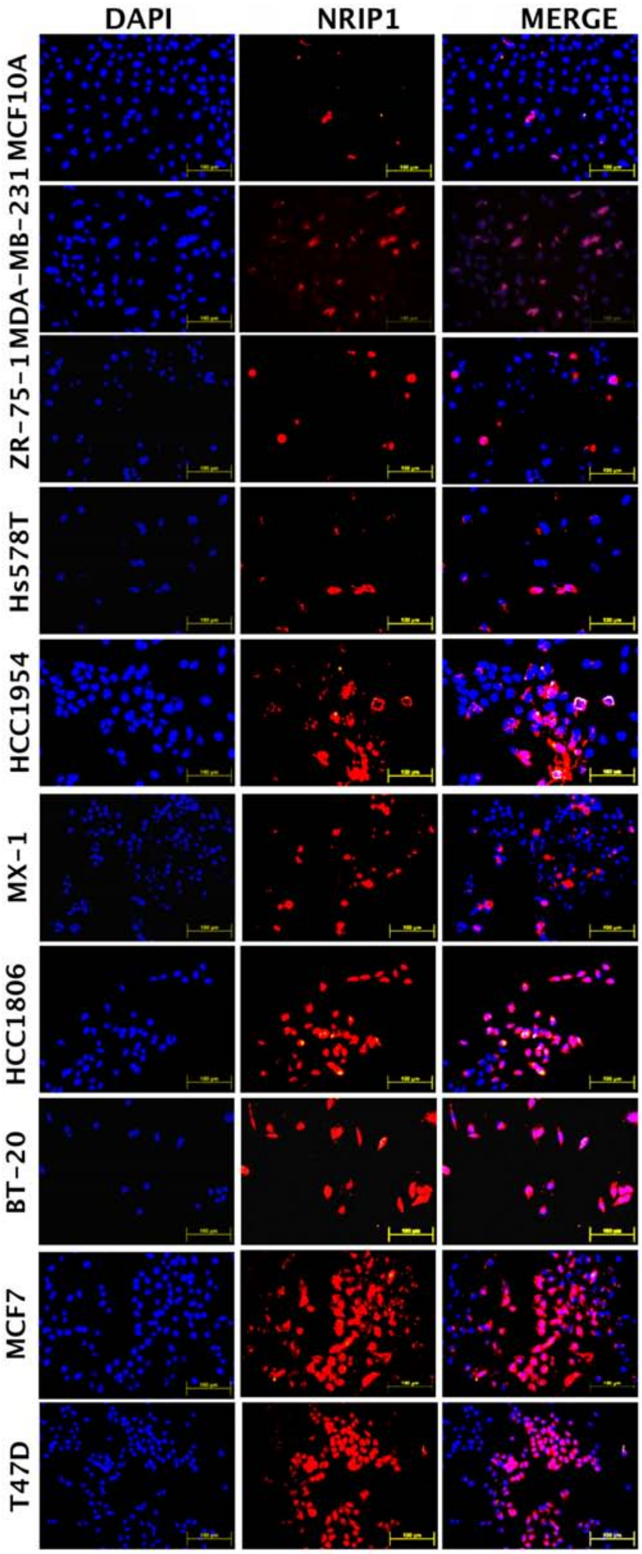

Supplementary Figure S1: NRIP1 level elevated in breast cancer cell lines. Immunofluorescence staining for NRIP1 was shown in breast cancer cell lines, normal immortalized breast cells (MCF10A). Representative images shown at 20X magnification with 100 µm scale bars.

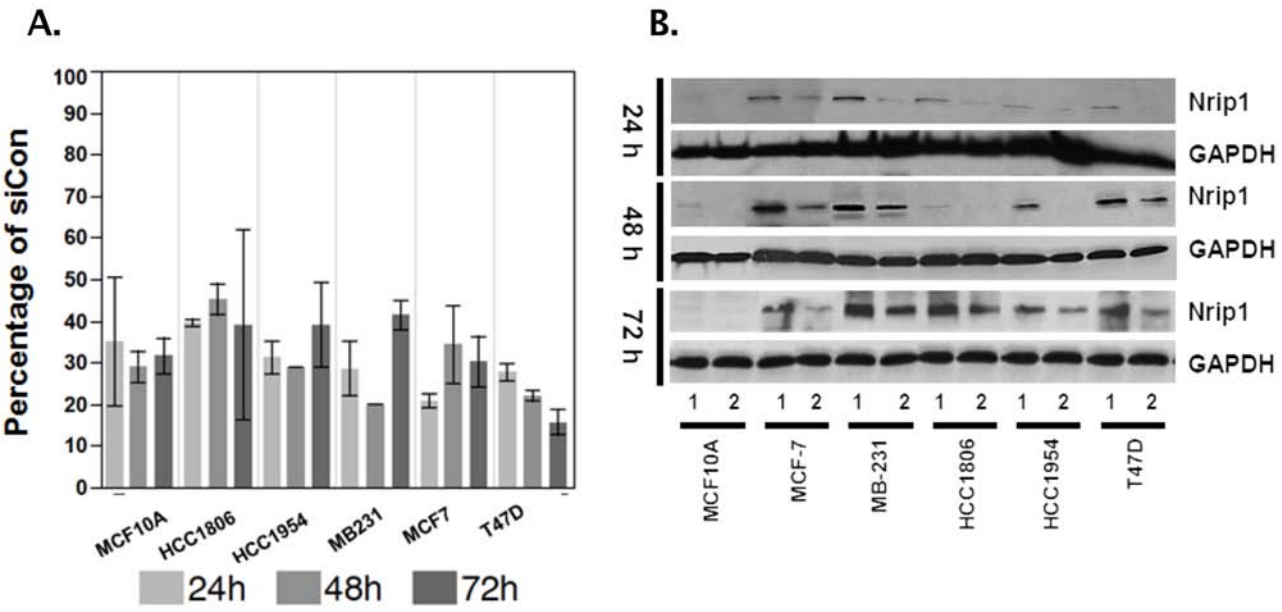

Supplementary Figure S2: Suppressing the expression of NRIP1 in breast cancer cell lines by siRNA. A. The knockdown efficiency is shown as 100*siRNA/siCON ($n \geq 2$). **B.** Western blot analysis at 24, 48 and 72 h after siNRIP1 and siCON treatment.

Supplementary Table S1: 24, 48 and 72 h PI staining

A. 24 h

	Apoptosis			G0/G1			S phase			G2/M		
**Cell lines**	**siCon**	**siNRIP1**	***p***	**siCon**	**siNRIP1**	***p***	**siCon**	**siNRIP1**	***p***	**siCon**	**siNRIP1**	***p***
MCF10A	3.2 ± 0.3	6.8 ± 0.8	0.02	77.4 ± 1.6	73.5 ± 3.2	0.34	3.8 ± 0.3	3.7 ± 1.2	0.90	15.4 ± 2.0	15.7 ± 1.8	0.92
HCC1806	3.6 ± 0.3	8.2 ± 0.3	<0.01	47.0 ± 0.3	44.0 ± 0.4	<0.01	12.2 ± 0.1	10.4 ± 0.3	<0.01	37.0 ± 0.2	37.5 ± 0.9	0.58
HCC1954	1.7 ± 0.1	6.6 ± 0.2	<0.01	44.9 ± 0.6	37.9 ± 0.4	<0.01	14.4 ± 0.7	16.5 ± 0.8	0.13	39.1 ± 0.2	39.7 ± 0.5	0.29
MB-231	2.4 ± 0.2	10.2 ± 0.4	<0.01	50.8 ± 0.3	42.7 ± 0.3	<0.01	10.5 ± 0.2	9.9 ± 0.4	0.29	36.3 ± 0.5	36.7 ± 0.4	0.55
MCF7	1.2 ± 0.0	5.6 ± 0.3	<0.01	48.8 ± 0.2	41.0 ± 0.5	<0.01	10.7 ± 0.2	10.3 ± 0.2	0.27	39.0 ± 0.2	42.8 ± 0.3	<0.01
T47D	2.0 ± 0.1	12.4 ± 0.4	<0.01	58.0 ± 0.8	57.4 ± 0.4	0.56	11.2 ± 0.1	8.3 ± 0.1	<0.01	29.5 ± 0.5	22.2 ± 0.5	<0.01
Overall	2.4 ± 0.4	8.3 ± 2.6	<0.01	54.5 ± 4.9	49.4 ± 5.5	0.02	10.5 ± 1.4	9.9 ± 1.7	0.45	32.7 ± 3.7	32.5 ± 4.4	0.85

B. 48 h

	Apoptosis			G0/G1			S phase			G2/M		
**Cell lines**	**siCon**	**siNRIP1**	***p***	**siCon**	**siNRIP1**	***p***	**siCon**	**siNRIP1**	***p***	**siCon**	**siNRIP1**	***p***
MCF10A	1.7 ± 0.1	4.9 ± 1.1	0.04	75.2 ± 0.1	71.5 ± 1.5	0.07	7.0 ± 0.9	7.0 ± 0.5	1.00	16.1 ± 0.9	16.1 ± 1.0	0.96
HCC1806	2.7 ± 0.3	10.9 ± 0.1	<0.01	51.3 ± 0.5	46.3 ± 0.3	<0.01	12.0 ± 0.3	13.7 ± 0.3	0.02	34.4 ± 0.3	29.7 ± 0.1	<0.01
HCC1954	3.0 ± 0.2	13.4 ± 0.2	<0.01	47.4 ± 0.2	37.7 ± 0.2	<0.01	14.2 ± 0.5	11.3 ± 0.1	<0.01	36.0 ± 0.4	38.3 ± 0.2	<0.01
MB-231	5.3 ± 0.2	21.5 ± 0.8	<0.01	48.2 ± 0.2	41.3 ± 0.3	<0.01	13.5 ± 0.2	10.9 ± 0.2	<0.01	33.0 ± 0.5	26.7 ± 0.4	<0.01
MCF7	1.8 ± 0.2	6.3 ± 0.9	<0.01	53.6 ± 0.4	38.7 ± 0.8	<0.01	11.1 ± 0.3	14.9 ± 0.7	<0.01	34.9 ± 0.6	39.7 ± 0.9	0.01
T47D	2.0 ± 0.0	9.8 ± 0.1	<0.01	59.3 ± 0.1	59.0 ± 0.4	0.51	5.9 ± 0.0	2.9 ± 0.1	<0.01	33.4 ± 0.3	28.9 ± 0.5	<0.01
Overall	2.8 ± 0.5	11.1 ± 2.4	<0.01	55.8 ± 4.3	49.1 ± 5.5	0.04	10.6 ± 1.4	10.1 ± 1.8	0.69	31.3 ± 3.1	29.9 ± 3.5	0.49

C. 72 h

	Apoptosis			G0/G1			S phase			G2/M		
**Cell lines**	**siCon**	**siNRIP1**	***p***	**siCon**	**siNRIP1**	***p***	**siCon**	**siNRIP1**	***p***	**siCon**	**siNRIP1**	***p***
MCF10A	3.1 ± 0.1	3.3 ± 0.8	0.81	76.0 ± 1.3	82.4 ± 1.0	0.02	7.7 ± 1.7	5.1 ± 0.5	0.23	13.8 ± 2.2	9.1 ± 1.0	0.12
HCC1806	3.0 ± 0.1	21.0 ± 0.5	<0.01	55.9 ± 0.2	48.0 ± 0.4	<0.01	8.9 ± 0.3	9.1 ± 0.5	0.68	32.8 ± 0.5	22.6 ± 0.7	<0.01
HCC1954	2.4 ± 0.1	12.3 ± 1.0	<0.01	44.3 ± 0.1	30.5 ± 2.3	<0.01	8.2 ± 0.3	17.8 ± 0.7	<0.01	45.7 ± 0.3	39.4 ± 2.7	0.08
MB-231	4.1 ± 0.3	13.0 ± 0.2	<0.01	46.6 ± 0.8	43.6 ± 0.2	0.02	15.9 ± 0.5	11.8 ± 0.4	<0.01	33.6 ± 0.5	32.0 ± 0.3	0.04
MCF7	1.3 ± 0.1	4.2 ± 0.1	<0.01	55.6 ± 0.6	51.0 ± 0.3	<0.01	15.6 ± 0.3	13.7 ± 0.7	0.08	27.7 ± 0.0	31.3 ± 0.5	<0.01
T47D	1.2 ± 0.0	9.2 ± 0.1	<0.01	59.4 ± 0.8	61.5 ± 0.4	0.07	7.6 ± 0.1	4.6 ± 0.2	<0.01	31.8 ± 0.3	22.8 ± 0.4	<0.01
Overall	2.5 ± 0.5	10.5 ± 2.7	0.02	56.3 ± 4.6	57.8 ± 7.2	0.11	10.6 ± 1.6	10.4 ± 2.1	0.94	30.9 ± 4.2	26.2 ± 4.3	0.14

Supplementary Table S2: Annexin-V analyses 24, 48 and 72 h after siRNA treatment

Time	Cell	siCon		siNRIP1		*p* value
		Mean	STE	Mean	STE	
24 h	MCF10A	2.1	0.5	3.8	0.1	0.07
	HCC1806	2.0	0.1	4.2	0.1	<0.001
	HCC1954	2.4	0.1	6.4	0.7	0.03
	MCF7	0.9	0.3	3.9	0.5	0.02
	MD-231	2.3	0.1	5.1	0.3	0.01
	T47D	3.0	0.1	6.7	0.4	<0.01
	Overall	2.1	0.3	5.0	0.5	<0.01
48 h	MCF10A	0.7	0.2	2.3	0.1	<0.01
	HCC1806	1.7	0.6	7.4	0.2	<0.01
	HCC1954	1.3	0.4	8.8	0.4	<0.001
	MCF7	1.2	0.1	4.0	0.3	0.01
	MD-231	2.0	0.5	7.5	0.2	<0.01
	T47D	1.8	0.2	12.3	1.3	0.01
	Overall	1.5	0.2	7.0	1.3	0.01
72 h	MCF10A	1.4	0.5	3.2	0.1	0.06
	HCC1806	1.5	0.3	10.6	0.5	<0.001
	HCC1954	1.9	0.1	10.4	0.6	<0.01
	MCF7	1.4	0.4	8.2	0.5	<0.001
	MD-231	1.3	0.1	5.3	0.3	<0.01
	T47D	1.8	0.3	13.9	0.2	<0.0001
	Overall	1.6	0.1	8.6	1.4	0.01
